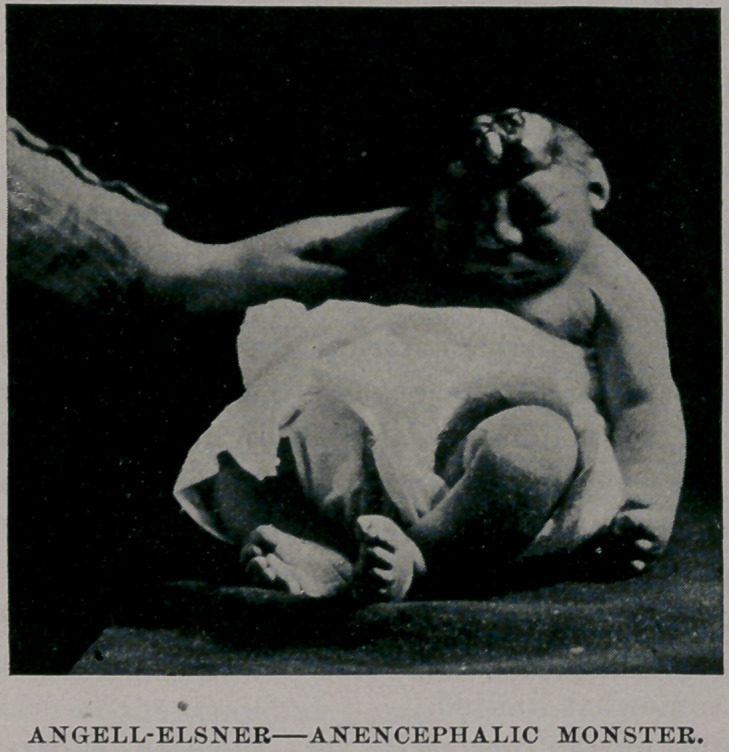# Observations upon an Anencephalic Monster1Read by Dr. Elsner before the Central New York Medical Association, Buffalo, N. Y., October 16, 1894.

**Published:** 1895-03

**Authors:** E. B. Angell, S. L. Elsner

**Affiliations:** Rochester, N. Y.; Rochester, N. Y.


### OBSERVATIONS UPON AN ANENCEPHALIC MONSTER.[1]

By E. B. ANGELL, M. D., and S. L. ELSNER, M. D., Rochester, N. Y.

ANENCEPHALUS is a monstrosity of not great rarity in a large obstetrical practice. It is, however, uncommon for delivery to occur at term and for the infant to live for any definite period. It was my misfortune to deliver such a monster in January last.

The mother, *æt.* 32 years, had given birth to three healthy children, all living. There was nothing noteworthy during the period of pregnancy, which was prolonged rather than shortened. The labor was a tedious one, the presentation difficult of determination and finally the forceps were employed. During the course of

1 Read by Dr. Elsner before the Central New York Medical Association, Buffalo, N. Y., October 16, 1894.

parturition the fetal movements were unusually violent, a circumstance often noted during the delivery of such monstrosities.

Aside from the head the infant, a male, was well proportioned, as will be seen by the photographs prepared for us by Dr. Weigel. The skull vault was absent, displaying a mass similar in shape and contour to the cerebrum and cerebellum, and occupying the central portion of the cranium. The longitudinal fissure is distinct, and to a fanciful observer even the rolandic fissures, as well as rudimentary convolutions, suggest themselves. At its posterior border were two distinct protuberances, which undoubtedly represented

ANGELL-ELSNER—ANENCEPHALIC MONSTER.

the cerebellum. The upper margin of the occipital bone had become inverted, almost occluding the posterior fossa. The above protrusions rested upon and were outside the bone. The whole mass was soft, semi-fluctuating, cystic, and somewhat nodular in character. It was covered by a vascular membrane, continuous at its base with the scalp, but devoid of hair. There was no pulsation, nor was there any change in tension during sleep or when the infant would cry. The following measurements were recorded:

DIAMETERS.

| | |
|---|---|
| Occipito frontal | 7.5 cm. |
| " mental | 8 2 " |
| Bi-temporal | 7 " |

| | | |
|---|---|---|
| Cervico-bregmatic ....... | 5 cm. | allowing 4 cm. for elevation of tumor. |
| Suboccipito-bregmatic ... | 7 " | |
| Fronto-mental ................ ........... | 7 | cm. |

CIRCUMFERENCES.

| | | |
|---|---|---|
| Head ...................................... | 28 | cm. |
| Tumor ..................................... | 18 | " |
| Bi-auricular arc ............................ | 12 | " |
| Transverse diameter (between shoulders) ..... | 22.8 | " |
| Length of child ............................. | 54.8 | " |
| Girth " chest ............................. | 40.7 | " |
| " " abdomen ............................. | 33 | " |
| Weight .................................... | 9 | lbs. |

The infant survived eight days and furnished us an opportunity for some interesting observations.

There was double divergent strabismus, and the limbs were in a state of constant spastic contraction. The reflexes, deep and superficial, were found to be exaggerated, the eye, or conjunctival reflex, also being present.

While there was double external strabismus, with partial ptosis and slight protrusion of the eyeball, the infant appeared able to direct its gaze to a limited extent, a lighted candle holding its attention. The pupils were in a mid-state between dilatation and contraction, but responded, though feebly, to stimulation.

Sensation of pain apparently persisted, a pin prick resulting in a wry face with a slight outcry, this undoubtedly being reflex rather than volitional. This sensory reaction was present everywhere, but somewhat limited.

The child nursed perfectly, this function apparently being instinctive or automatic. In fact, simple reflex action plays a more important role in the matter of mere existence than is generally credited.

Pressure upon the nodules of the pseudencephalic mass produced a convulsive spasm of the muscles generally. On the second day stimulation by the faradic current, with slow interruption, was resorted to. At first, its application being restricted to the surface of the tumor, metal electrodes were applied, one centimeter apart, to the supposed motor area, but even with a strong current no reaction was obtained. The electrodes were then gradually separated until the whole tumor was included, without any muscular effect. By increasing the current, strong contraction was finally developed in the neighboring scalp muscles. At no time did electric stimulation of what appeared to be the motor area of the cortex, develop reaction in the corresponding muscles, although on the sixth day an insulated gold needle was introduced within the structure of the tumor, as a sub-cortical electrode. Puncture by the needle, however, gave escape to a quantity of bloody serum. On the seventh day both sides of the mass were aspirated, about three drachms of fluid being withdrawn. This operation was not attended by any inflammatory action or rise of temperature.

From the second or third day occasional convulsions occurred and after the aspiration they became more frequent and severe, the infant dying in a hard seizure upon the eighth day. Owing to the extreme prejudices of the family, no satisfactory autopsy could be secured and a hurried examination of the head had to suffice. The skull was perfectly flat on a plane through the junction of the nasal and supraorbital arch of the frontal and the superior angle of the occipital bone. There was no frontal bone, beyond a rudimentary orbital plate. The parietal bones were wholly absent, the base of the tumor occupied fully two-thirds of this flat surface, the circumference at the base being 17.8 cm., while the skull at the same level measured only 28 cm. The tumor measured 6.2 cm. longitudinally, 5.1 cm. transversely and had an elevation of 4 cm.

Upon dissecting the tumor from its bed, its under surface was found to rest upon a flat, bony floor, made up of the cribriform plate of the ethmoid, the body and large wings of the sphenoid, the bassilar portion of the occipital bone and inverted edges of the squamous portion of the temporal bones.

The optic and one or two other cranial nerves were found upon the floor of the skull. There was no anterior or middle fossa. The opening into this shallow skull, through which this mass connected with the medulla, was located just posterior to the basilar portion of the occipital bone. This opening was about 1.3 cm. in diameter and led posteriorly into a small fossa and thence downward directly to the foramen magnum.

The occipital bone was complete, but its development so greatly retarded that it afforded no room for the cerebellar nodules which rested upon its inverted margin. This cavity was only partially occupied by the shrunken pons. The occipital bone being united and the spinal column preserved, the monster was an anencephalus or pseudencephalus of the nosencephalic type according to the classification of Geoffrey St. Hilaire.

Dissection of the mass was impossible, but macroscopically in place of the normal structure it seemed largely composed of bands or strands of connective tissue, surrounding cystic prolongations of the cerebral cavities.

Upon tearing out the few shreds of cortical tissue we smuggled away, a microscopical examination disclosed a few scattered ganglion cells with a marked predominance of connective tissue.

## Figures and Tables

**Figure f1:**